# The Implementation of a Virtual Emergency Department: Multimethods Study Guided by the RE-AIM (Reach, Effectiveness, Adoption, Implementation, and Maintenance) Framework

**DOI:** 10.2196/49786

**Published:** 2023-12-05

**Authors:** Jennifer Shuldiner, Diya Srinivasan, Laura Desveaux, Justin N Hall

**Affiliations:** 1 Women's College Hospital Institute for Health System Solutions and Virtual Care Women's College Hospital Toronto, ON Canada; 2 Trillium Health Partners Mississauga, ON Canada; 3 Department of Emergency Medicine Sunnybrook Health Sciences Centre Toronto, ON Canada; 4 Division of Emergency Medicine Temerty Faculty of Medicine University of Toronto Toronto, ON Canada; 5 Institute of Health Policy, Management and Evaluation University of Toronto Toronto, ON Canada

**Keywords:** emergency care, virtual care, implementation science, mixed methods evaluation, emergency department, pilot, evaluation, triage, digital emergency department

## Abstract

**Background:**

While the COVID-19 pandemic dramatically increased virtual care uptake across many health settings, it remains significantly underused in urgent care.

**Objective:**

This study evaluated the implementation of a pilot virtual emergency department (VED) at an Ontario hospital that connected patients to emergency physicians through a web-based portal. We sought to (1) assess the acceptability of the VED model, (2) evaluate whether the VED was implemented as intended, and (3) explore the impact on quality of care, access to care, and continuity of care.

**Methods:**

This evaluation used a multimethods approach informed by the RE-AIM (Reach, Effectiveness, Adoption, Implementation, and Maintenance) framework. Data included semistructured interviews with patients and physicians as well as postvisit surveys from patients. Interviews were transcribed and analyzed using thematic analysis. Data from the surveys were described using summary statistics.

**Results:**

From December 2020 to December 2021, the VED had a mean of 153 (SD 25) visits per month. Among them, 67% (n=677) were female, and 75% (n=758) had a family physician. Patients reported that the VED provided high-quality, timely access to care and praised the convenience, shorter appointments, and benefit of the calm, safe space afforded through virtual appointments. In instances where patients were directed to come into the emergency department (ED), physicians were able to provide a “warm handoff” to improve efficiency. This helped manage patient expectations, and the direct advice of the ED physician reassured them that the visit was warranted. There was broad initial uptake of VED shifts among ED physicians with 60% (n=22) completing shifts in the first 2 months and 42% (n=15) completing 1 or more shifts per month over the course of the pilot. There were no difficulties finding sufficient ED physicians for shifts. Most physicians enjoyed working in the VED, saw value for patients, and were motivated by patient satisfaction. However, some physicians were hesitant as they felt their expertise and skills as ED physicians were underused. The VED was implemented using an iterative staged approach with increased service capabilities over time, including access to ultrasounds, virtual follow-ups after a recent ED visit, and access to blood work, urine tests, and x-rays (at the hospital or a local community laboratory). Physicians recognized the value in supporting patients by advising on the need for an in-person visit, booking a diagnostic test, or referring them to a specialist.

**Conclusions:**

The VED had the support of physicians and facilitated care for low-acuity presentations with immediate benefits for patients. It has the potential to benefit the health care system by seeing patients through the web and guiding patients to in-person care only when necessary. Long-term sustainability requires a focus on understanding digital equity and enhanced access to rapid testing or investigations.

## Introduction

Overcrowding and wait times within emergency departments (ED) are persistent problems across health systems. In Ontario, Canada, visits to EDs have increased by 24.8% in the last decade [1,2]. As ED visits increase and wait times to physician assessment lengthen, resources are strained, and patient satisfaction decreases [3]. COVID-19 has propelled telehealth beyond the phase of early adoption into a substantial component of health systems in Canada and abroad [4,5]. At the outset of the COVID-19 pandemic, EDs worldwide faced a substantial decrease in patients seeking in-person emergency care signaling the need for web-based or phone services [6]. As a result, there is growing interest in identifying alternative patient care options to enhance system sustainability [7,8] and optimize resource use.

Novel treatment pathways that use innovative deployment of technology hold promise for meeting rising patient demand without compromising high-quality care [9] and are the focus of many professional and regulatory associations [10]. Some systems have explored using technology for low-acuity patients to reduce overcrowding and increase access [11-16]. Despite this, the reluctance of the medical community to adapt to new technology and uncertainty around how to adopt existing workflows have precluded significant advancements [17]. In parallel to rising patient support and enthusiasm in Canada [18], health system stakeholders expect that if appropriately applied, telemedicine technology will improve health care access and allow the delivery of high-quality care at reduced cost [9,19].

In response to these realities, Sunnybrook Health Sciences Centre (SHSC), an academic quaternary-care hospital in Toronto, Ontario, developed the region’s first virtual emergency department (VED) that connected low-acuity patients directly to ED physicians. The design, planning, and implementation were informed by patients and providers of virtual care services in other specialties including internal medicine, family practice, oncology and emergency services in other regions. Key recommendations included having a user-friendly booking system for appointments, provider training on how to deliver virtual care including physical examination maneuvers, and dedicated administrative support for both patients and providers. To support iterative developments and understand sustainability needs, we conducted an embedded evaluation informed by the RE-AIM (Reach, Effectiveness, Adoption, Implementation, and Maintenance) framework [20,21]. The objectives of the evaluation were to (1) assess the acceptability of the VED model at SHSC, (2) evaluate whether the VED was implemented as intended, and (3) explore the impact on quality of care, access to care, and continuity of care.

## Methods

### Study Design

We conducted a multimethods study guided by the RE-AIM framework to evaluate the implementation of the VED [20,21] (Table 1). The RE-AIM framework is a planning and evaluation model that focuses on 5 elements of individual- and setting-level outcomes essential to program impact and sustainability: Reach, Effectiveness, Adoption, Implementation, and Maintenance. Reach refers to the absolute number, proportion, and representativeness of individuals who participate in the program. Effectiveness is the impact of a program on important outcomes. Adoption is the absolute number, proportion, and representativeness of settings and intervention agents who initiate a program. Implementation refers to the intervention agents’ fidelity to and adaptations of intervention and associated implementation strategies, including consistency of delivery as intended. Finally, maintenance is the extent to which a program or policy becomes institutionalized or part of routine organizational practices and policies.

Data collection included surveys completed by patients of the VED and interviews with both patients and ED physicians. Data were simultaneously collected, analyzed separately, and then triangulated to obtain a deeper understanding of the RE-AIM dimensions.

**Table 1:** RE-AIM (Reach, Effectiveness, Adoption, Implementation, and Maintenance) dimensions that were explored in the multimethods evaluation of the Sunnybrook Health Sciences Centre virtual emergency department (VED).

Dimension	Key questions that were considered	Data collection
Reach	Who are the patients who accessed the VED?	Metrics on the number of patient visits and survey data on patient population
Effectiveness	How has the VED impacted quality of care, continuity of care, and access to care?	Patient survey data and interviews with patients
Adoption	How has the VED been adopted by emergency department physicians?	Interviews with physicians
Implementation	How has the VED been implemented? How has it been adapted?	Interviews with physicians and patients
Maintenance	Did the VED transition out of a pilot? How long will it be sustained?	N/A^a^

^a^N/A: not applicable.

### Context and Setting

SHSC is an adult academic quaternary care hospital in Toronto, Ontario, affiliated with the University of Toronto. It is a regional trauma, oncology, neonatal, high-risk maternal, neurosurgical, interventional cardiology, and stroke center. The hospital sees 1.3 million patient visits and 62,000 ED visits annually. ED physicians are funded through an Alternate Funding Agreement with the Ontario Ministry of Health and Long-Term Care [22].

### The Intervention

SHSC launched a 6-month VED pilot in December 2020, which was subsequently extended and continues to operate. During the initial pilot, ED physicians staffed the VED on weekdays from 2 to 9 PM, in parallel to the in-person ED running at normal capacity. The VED was staffed by a single physician at a time, and the physician’s shift was solely dedicated to seeing virtual patients (ie, no in-person patients from the regular ED were seen by the VED physician on duty).

**Figure 1:**
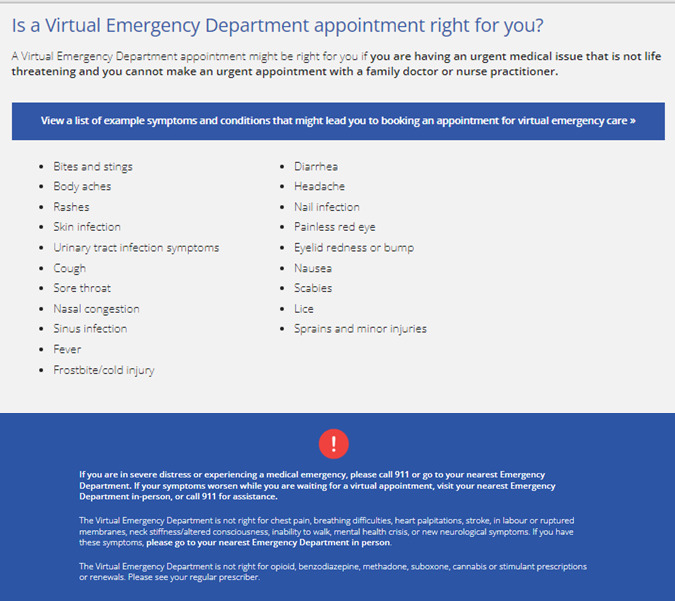
The Sunnybrook Health Sciences Centre virtual emergency department list of appropriate and inappropriate conditions for a virtual visit along with advice of when to consult their family physician as their primary contact for lower acuity concerns.

Patients access the VED through the SHSC website and self-triage using a web form. A standard informational message informs patients of potentially appropriate and inappropriate conditions for a virtual visit along with advice of when to consult their family physician (Figure 1). Once patients determine that their visit is appropriate, they can then register on the web for a same-day appointment at the VED. A dedicated patient administrative associate confirms demographic information, validates their health card, and creates an appointment that is emailed or texted to the patient. The patient then clicks on the Zoom (Zoom Video Communications) appointment link at the time of their visit and connects with an ED physician. Following completion of the VED visit, it is classified into one of the following five outcomes: (1) care has been fully managed during the virtual appointment including potential prescriptions, (2) referred to management by their family physician, (3) referred to a specialist, (4) scheduled for follow-up for diagnostic imaging such as x-rays or ultrasounds, or (5) requested to attend the ED in person for further management. The visit is part of the Sunnybrook electronic health record system, with all records shared with the provincial Health Report Manager and Connecting Ontario systems for access by family physicians and specialists outside of the organization.

The VED was implemented using an iterative staged approach with increased service capabilities over time, including access to ultrasounds, virtual follow-ups after a recent ED visit, and access to blood work, urine tests, and x-rays (at the hospital or a local community laboratory).

### Recruitment and Data Collection

#### Patient Surveys

The survey was developed by researchers based on a review of relevant literature and discussion with virtual urgent care providers, qualitative methodologists, and a clinical epidemiologist. The questionnaire was peer-reviewed by 5 people unrelated to the study and tested for face and construct validity as well as comprehension. The survey includes multiple choice, Likert, and open-ended questions regarding how the patient heard of the VED, why they chose the VED, the availability of a primary care provider, and their perceived experience and satisfaction with the VED. The survey included questions regarding quality of care, patient satisfaction, continuity of care, and economic impact regarding their ED visit. Survey instruments aligned to recommended domains of measurement that can be used across evaluations of digital health solutions and virtual care according to the National Quality Forum [23], were reviewed with patients and providers to establish validity and reliability, and were piloted (Multimedia Appendix 1). An invitation to complete a web-based survey was emailed to all patients who had a visit at the VED through a link using REDCap web-based software (Research Electron Data Capture; Vanderbilt University). The data are anonymous and cannot be linked back to the patient. Participants were not required to answer every question, and each question had a different number of responses.

#### Physician Interviews

Physicians who participated in the VED received an invitation via email from the implementation lead of the VED (JNH). Physicians who wanted to participate contacted the study coordinator who explained the study, provided a study information sheet, and obtained consent. All interviews were conducted by a qualitatively trained researcher (DS) and were recorded and transcribed verbatim. We collected descriptive information such as demographic details (ie, gender and age) and data on their ED experience (ie, number of shifts worked per month). Interviews explored overall provider experience, how the VED may have impacted access to care, continuity of care, and overall care, and the implementation of the VED.

#### Patient Interviews

Interviews were conducted by DS with patients after their VED visit. The managing physician or administrative personnel from SHSC asked the patient if they consent to being contacted for the purposes of evaluation. A detailed information sheet was provided to the patient. If the patient agreed to be contacted, the patient's email address was provided to the research team. Interviews aimed to gain a deeper understanding of how their VED encounter impacted access to care, continuity of care, and overall care. We invited participants with diverse VED experiences (ie, who went through different care pathways and who had varying reasons for a visit). Participants who consented to and completed the interview were offered a CAD $30 (US $21.91) compensation as a “thank you.”

### Data Analysis

#### Patient Surveys

Using data collected from REDCap, descriptive summary statistics (means, SDs, frequency counts, and percentages) were calculated. Data are organized and presented by the RE-AIM components.

#### Patient and Physician Interviews

Thematic analysis using inductive coding was used to describe the manifest and latent content [24]. The first 3 transcripts were coded independently line-by-line by 2 research team members (DS and JS), and codes were compared for the first 3 interviews to achieve consensus and establish a codebook. The remaining interviews were coded independently by a single member of the research team (DS) using the codebook as a guide. The first step in analyzing the codes and identifying themes was to deductively map codes to RE-AIM domains. Subsequent levels of coding involved reexamining the content of the codes and narrowing in on more common elements discovered in the data during coding. Initial themes were then reviewed with the full study team and refined to ensure that the themes represented the data set as a whole and that no themes were missed or overrepresented. Data collection and analysis continued iteratively until saturation was achieved, that is, until no new ideas were introduced during subsequent interviews [25].

### Ethical Considerations

Ethics approval was received from the SHSC Research Ethics Board for the survey (REB #: 3654). The survey included a statement “I consent to my responses being used for research purposes” so patients can decide if they do not want their answers to be used for research purposes. For the interviews, this initiative was formally reviewed by institutional authorities at Women’s College Hospital and was deemed not to require Research Ethics Board approval (REB #2021-0040-E). Regarding the interviews, the research coordinator explained the purpose of the evaluation, study activities, and risks and then obtained consent. The survey and interview data were deidentified before analyses. Data from digital recordings were kept on a secure server and were deleted once transcribed and verified for accuracy. Amazon gift cards were given to patients (CAD $30 [US $21.91]) and physicians (CAD $50 [US $36.51]) in appreciation for interviewees’ time.

## Results

### Overview

A total of 176 patients responded to the patient experience survey (Multimedia Appendix 2) and agreed for their responses to be used for research. Interviews were completed by 15 patients (Table 2) and 14 providers (Table 3).

**Table 2:** Characteristics of patients who participated in a Sunnybrook Health Sciences Centre virtual emergency department qualitative interview (N=15).

Characteristics				Values
**Sex, n (%)**				
	Female		9 (60)	
	Male		6 (40)	
	Other		0 (0)	
**Education, n (%)**				
	High school		5 (33)	
	University		6 (40)	
	College or diploma		2 (13)	
	Doctorate degree		1 (6)	
	Unknown		1 (6)	
Age (years), mean (SD)				51 (26)
**Primary language, n (%)**				
	English		14 (93)	
	Other		1 (7)	
**Children, n (%)**				
	Yes		9 (60)	
	No		4 (27)	
	Unknown		2 (13)	
**Marital status, n (%)**				
	Married or common law		8 (53)	
	Single		2 (13)	
	Widowed		1 (7)	
	Divorced		2 (13)	
	Unknown		2 (13)	
**Ethnicity or cultural identity, n (%)**				
	Caucasian or Canadian		9 (60)	
	Other		4 (27)	
	Unknown		2 (13)	
**Refugee or immigrant, n (%)**				
	Yes		2 (13)	
	No		11 (74)	
	Unknown		2 (13)	
**Family physician visit frequency, n (%)**				
	0-1 per year		6 (40)	
	2-4 per year		4 (27)	
	5 or more per year		3 (20)	
	Unknown		2 (13)	
**Last visit to family physician, n (%)**				
	Last week		4 (27)	
	2+ weeks to 12 months		8 (53)	
	More than 1 year		1 (7)	
	Unknown		2 (13)	
**Overall health rating, n (%)**				
	Excellent		3 (20)	
	Very good		2 (13)	
	Good		5 (34)	
	Fair		3 (20)	
	Poor		0 (0)	
	Unknown		2 (13)	
**Diagnosis, n (%)**				
	**Acute**			
		Skin and soft tissue	4 (27)	
		Orthopedics	2 (13)	
		Obstetrical-gynecological	1 (6)	
		Respiratory	1 (6)	
		Trauma and injury	1 (6)	
		Cardiovascular	1 (6)	
		Genitourinary	1 (6)	
		Otolaryngology	1 (6)	
	**Acute or chronic**			
		Acute	3 (20)	

**Table 3:** Characteristics of emergency department physicians who participated in a Sunnybrook Health Sciences Centre virtual emergency department qualitative interview (N=14).

Characteristics		Values
Age (years), mean (SD)		46 (10)
**Sex, n (%)**		
	Male	7 (50)
	Female	7 (50)
Experience (years), mean (SD)		16 (11)

### Reach

From December 2020 to December 2021, the VED had a total of 1987 virtual visits from 1010 unique patients with a mean of 153 (SD 25) visits per month. Among them, 67% (n=677) were female, and 75% (n=758) had a family physician (Table 4). The top 3 chief presenting complaint categories were orthopedic, gastrointestinal, and skin and soft tissue.

A total of 176 patients responded to the patient experience survey and agreed for their results to be used for research out of 1010 patients who completed a visit from April 12, 2021, to November 22, 2021 (17.4% response rate). Most patients had a family doctor (n=189, 93%), completed postsecondary education (n=144, 86%), spoke English as a primary language (n=151, 87%), and were in good health (n=134, 75%; Table 5). The most common reason for choosing the VED was the perception that it was most appropriate (n=72, 35%), followed by an inability to make an appointment with their family doctor (n=59, 30%), or they received direct guidance from their family doctor to go to ED (n=18, 8%). The VED was used as an alternative to an in-person ED visit for 70% (n=107) of respondents. For most patients (n=120, 58%), this VED visit was their first experience with virtual care. Most patients’ main reason for accessing the VED was due to a new health problem (n=102, 49%), followed by an ongoing health concern (n=66, 32%) or an injury (n=39, 19%).

**Table 4:** Characteristics of patient visits to the SHSC^a^ virtual emergency department from December 2020 to December 2021 (N=1987).

Characteristics			Values, n (%)
**Sex**			
	Female	1321 (67)	
	Male	666 (33)	
**Age (years)**			
	<18	87 (4.4)	
	18-34	630 (31.7)	
	35-50	620 (31.2)	
	51-64	326 (16.4)	
	65-79	235 (11.8)	
	80+	89 (4.5)	
Have a family physician			1480 (74.5)
**Disposition**			
	Home	811 (40.9)	
	ED^b^	529 (26.6)	
	Specialist referral	336 (16.9)	
	Back to family physician	286 (14.4)	
	No show	24 (1.2)	
Nearest ED: SHSC			588 (29.6)
Live within Toronto Central Local Health Integration Network			936 (47.1)

^a^SHSC: Sunnybrook Health Sciences Centre.

^b^ED: emergency department.

**Table 5:** Characteristics of patients who completed the Sunnybrook Health Sciences Centre virtual emergency department cross-sectional evaluation survey (N=176).

Characteristics		Values
**Sex, n (%)**		
	Female	122 (69.3)
	Male	50 (28.4)
	Other	4 (2.2)
**Education, n (%)**		
	Elementary school	3 (1.8)
	High school	24 (14)
	College diploma	19 (11.1)
	Bachelor degree	58 (33.9)
	Graduate degree	46 (26.9)
	Professional school	21 (12.3)
**Primary language, n (%)**		
	English	151 (86.8)
	Other	23 (13.2)
Time to get to the ED^a^ (minutes), mean (SD)		27.2 (31.4)
Average distance to the ED (km), mean (SD)		11.6 (14.2)
**Have a family doctor, n (%)**		
	Yes	189 (92.6)
	No	15 (7.4)
**Overall health, n (%)**		
	Excellent	29 (16.6)
	Very good	54 (30.9)
	Good	51 (29.1)
	Fair	31 (17.7)
	Poor	10 (5.7)

^a^ED: emergency department.

### Effectiveness

The survey results indicated that the VED was perceived to be effective across the domains of quality of care, continuity, and access from a patient perspective. Patients praised the convenience, shorter appointments, and the benefit of a calm, safe space that can be accessed through virtual appointments.

I was scared of COVID. I didn't want to wait eight hours and it's just so efficient. It's just such a great way to do an emergency appointment, because you have an appointment and you don't have to wait so long, and wonder when you're going to be seen ... There's no parking, there's no wait time. It's extremely efficient. You get the one-on-one personal attention with no distractions.

Patient 8

Patients felt that VED allowed them to receive quality care in a timely (n=178, 89%) and convenient (n=179, 89%) manner and provided useful information (n=166, 83%) with high satisfaction (n=185, 90%). Most patients were comfortable connecting by phone call or video call (n=200, 93%), felt their privacy was respected (n=188, 93%), and had sufficient time during their visit (n=185, 90%).

In the interviews, patients described how they experienced quality care on the VED platform. Patients felt that physicians were effective in how they communicated during their visit. The video component was cited as invaluable and differentiated SHSC's VED from other telehealth platforms.

The appointment felt as if I was in the office or at the hospital. He was attentive, asked me appropriate questions. And I was able to move my camera so he could see my leg and assess it virtually, which was nice. I found him to be very thorough. He didn't just brush me off and listened to my concerns. I actually had to go back to emerge. So, that was clear and he was very compassionate about it all. Virtual care experience was great.

Patient 2

Patients appreciated the undivided attention of the physician. VED appointments felt more comfortable than in-person, as patients felt the physician was more distracted in the in-person ED and the environment itself is noisy and potentially chaotic.

I felt like I had his undivided attention because there's no distractions. In the emergency room, there's always distractions with noises and things. But over the computer, it was quiet on both ends and I had his complete attention.

Patient 3

Most patients felt that physicians understood their health concerns as much as possible (n=164, 81%), had a clear understanding of their health concerns (n=150, 73%), and had all the information they needed (n=140, 68%). These sentiments were also echoed in the patient interviews:

Yeah, it worked really well for me. It literally saved me a whole day and the experience was good. The doctor was very understanding. We had a conversation about my symptoms about where I was in my recovery. And they seemed confident that I didn't need to do anything immediately, and it worked out.

Patient 5

Many patients wanted to know if their concerns were valid and if they warranted an in-person ED visit. Among our interviews, we found that the VED helped to direct 2 patients for an in-person visit, and many patients away from it.

My daughter actually was the one that set it up, because we didn't know what to do, whether we should go to the hospital or not. And so, it was basically under that decision on talking to the doctor that she told us, we better take him to the hospital.

Patient 6

The virtual appointments also provided a sense of safety from the ongoing pandemic and a sense of clarity around the patient’s health concern, and it gave confidence to those who required an in-person visit that they should be there:

It was a very good thing [to have the virtual emergency department visit] because we felt when we went [to the physical emergency department] that we should be there. Because going to emergency during the pandemic, you don't really like to go unless it's really important.

Patient 6

Continuity of care was maintained for most patients, and 68% (n=135) felt confident they could manage their health issues at the end of the visit, and 73% (n=135) left the visit with a care plan (see “The Intervention” section for possible visit outcomes).

Approximately one-third of patients (n=65, 32%) did not need to access additional care following the VED visit. The remainder of the patients were either given an appointment with another health care provider (n=51, 25%), advised to follow-up with their family physician (n=34, 17%), told to go to the ED (n=40, 19%), or given an appointment at the hospital for imaging (n=15, 7%).

Almost everyone who received advice to visit the in-person ED or was scheduled for imaging at the hospital honored their appointments (n=37, 95%). In contrast, a slightly lower percentage attended appointments with other health care providers (n=40, 78%). Only 32% (n=11) of those who were advised to follow-up with their family physician reported that they did so.

In the interviews, most patients noted that regardless of postvisit trajectory, the process was clear. There were mixed reports on the shift from the VED to the in-person ED. Some felt better equipped and mentally prepared for their visit, while other patients found that there was some repetition from the VED to the in-person ED and would have appreciated more integration.

Patients who were referred directly for an ultrasound or an appointment described a smoother transition as they were able to circumvent the in-person ED:

So it was fantastic. My dad got antibiotics, he [the physician] was able to diagnose over the phone. There was a follow up ultrasound already booked for my dad for the next morning with instructions in the emergency room to be assessed there to see if any further treatment would be needed.

Patient 14

Finally, another reported benefit was about 60% (n=110) of respondents agreed that the VED saved them or their caregiver from out-of-pocket costs and resulted in decreased time away from work. Patients were overwhelmingly impressed by the impact on access to care and how quickly they received appointments:

The pros is that I got an appointment ASAP, which is amazing. It was right away. And as I said, the first experience with my mum was exceptional. I mean, it was just straightforward and we did go to the hospital, we did what we needed to do.

Patient 7

Two-thirds of patients reported that if the VED was not available, they would have gone to the physical ED (n=107, 62%), with the remaining one-third (n=58, 28%) reported they would go to their family physician. Many patients (n=69, 29%) reported their reason for the VED visit was that they either could not be seen or could not get an appointment soon enough with their family physician. In the absence of the VED, patients reported they would have to travel a mean time of 27 (SD 31) minutes and a mean distance of 12 (SD 14) km to visit their in-person ED.

### Adoption

**Figure 2:**
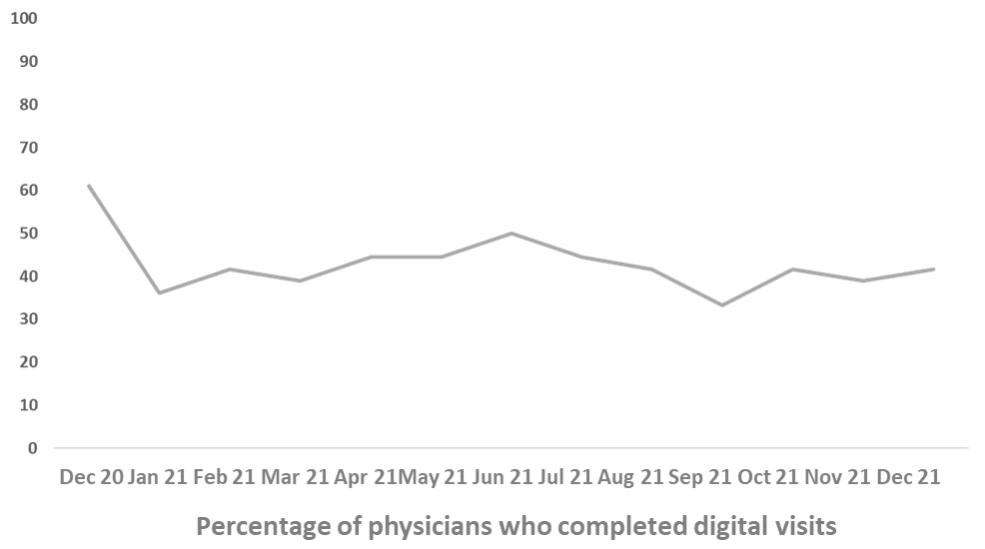
Percentage of emergency department physicians who sustained the Sunnybrook Health Sciences Centre virtual emergency department engagement from December 2020 to December 2021.

There was a broad uptake of VED shifts among ED physicians with most physicians completing more than 1 shift per month. At the beginning of the pilot, about 60% (n=22) of physicians were completing virtual shifts, and that number dropped to 42% (n=15) about a year later with a mean of 42% (SD 7) completing virtual shifts throughout the first year of the pilot (Figure 2). There were no difficulties finding sufficient ED physicians for shifts, and some providers were able to engage in these shifts if they had to self-isolate due to COVID-19 exposures. The virtual shifts were spread among physicians to ensure all those who requested shifts would have the opportunity to complete at least 1 monthly.

Many physicians had concerns about the quality of care within a VED model in its early stages. For some, these were alleviated when the value for patients and physicians became clear. Most physicians enjoyed working in the VED, saw value for patients, and were motivated by patient satisfaction. However, some physicians were hesitant as they felt their expertise and skills as ED physicians were underused. These physicians described their belief that any health issue that did not require in-person care was an issue meant for a primary care physician. The virtual model limited their ability to use what they believed to be key resources of the ED, which led some to feel unfulfilled.

I don't think that what we're doing is specific to emergency medicine. Like the job that we signed up for, for emerge is more the acute things that day-to-day stuff with resources ... I don't feel that the virtual emerge is fulfilling to me in terms of being an emergency physician.

Physician 8

Conversely, we found that many physicians witnessed the value of the VED first-hand and found their shifts were a meaningful use of their time, overcoming early skepticism.

Initially I was quite sceptical as to how it would work, I've never really done virtual care before, and I couldn't envision patients that I would be able to treat virtually. I thought that any patient would wind up being sent into the emergency department for an evaluation ... But now having done it, I actually like it a lot and it does seem useful for patients.

Physician 15

Physicians found the VED valuable because they were able to support patients by advising on the need for an in-person visit, booking a diagnostic test, or a referral to a specialist. They also reported that they were able to use their knowledge and skills as an ED physician to help their patients:

When you physically work in an Emerge, you know the nuances of the system. So, I can do things a nurse can't, like send a referral or tell you what is the follow up process. Or I can tell you, you know if you come into the Emerge now, you're not going to get that ultrasound. They're not open until the morning anyway. You might as well just wait till the morning. Those are the kind of nuances I think that only the person who works there would be able to tell you and those are really valuable.

Physician 4

### Implementation

The patient survey showed that nearly all visits were conducted by video (n=201, 98%) with 6 (2%) experiencing technical issues that resulted in an audio-only consultation over the phone. Patients reported being able to explain their health issues (n=194, 93%), having adequate time with the health care provider (n=185, 91%), and intending to use virtual care again (n=191, 92%).

The VED rollout happened in stages, where the capabilities of the physicians increased with time. After a few months, physicians were able to book patients for ultrasound appointments at the hospital with in-person ED or outpatient follow-up. After 6 months, laboratory requisitions for same or next-day blood work or urine tests and x-rays were available for physicians to schedule.

Physicians described that as the capabilities of the VED expanded, their satisfaction and sense of providing good care increased:

And now we’ve expanded it to involve some basic blood tests and x-rays and ultrasounds that are available the next day ... And potentially almost act as a pre-triage to patients that might ultimately need to come into the emergency, but we could arrange the majority of their care before they get there.

Physician 12

Patients who were interviewed reported that the website was user-friendly and easy to understand and follow. They appreciated the confirmation email, and the instructions were clear. For those who were having trouble, administrator support was crucial for a smooth implementation. The administrator was available to troubleshoot any issues and help with registration when issues came up.

### Maintenance

Physician interviews indicated that many of them were satisfied and happy with their work with the VED and the care they were able to provide patients. This made them feel motivated about the work that they were doing and renewed their interest to complete more virtual shifts:

I feel stronger about it, now that I see that people are calling in. They have trust in the care that we're giving. Some people are calling just to get reassurance that they're OK to stay at home. Some people are calling to get treatment over the virtual emergency care platform ... we're providing successful care and people are pleased with the care they're receiving. So, we should be doing it ... That's a really great feeling is that I'm coming in, I'm doing a really, really positive shift, helping, that for myself as well, is positive. And I think that is important for sustainability as well. You want your physicians to be feeling good about the care they're giving.

Physician 2

There was a general sense that this program was beneficial and should be sustained as virtual care continues to be a significant component of health care delivery:

I mean I think it's a great initiative. It's long-time coming. Things got expedited because of the pandemic, from an IT and legal perspective, which has been fantastic. And like I said, I don't think this is going anywhere anytime soon.

Physician 5

Physicians were keen to see the VED continue as they had the sense that patients were satisfied and that they were helping ease the burden of the in-person ED:

I know patients really like it which is great and they're super thankful and so I think having more physicians available for them and also deterring patients from coming to the emergency department when they actually don't need to come is definitely a helpful thing for both the patients and the doctors who are working. So I do think there's a role for it going forward. As long as they are appropriate patients that are showing up then I think it's totally fine to continue with it because it will help alleviate some of the burden in the emergency department. And also [help increase] patient satisfaction for not having to wait five hours to get that referral to dermatology or ophthalmology.

Physician 8

## Discussion

### Principal Findings

This study evaluated the implementation of the SHSC VED according to the domains of the RE-AIM framework. The results found that patients and physicians viewed the VED as highly effective and beneficial. Key benefits included timely access to care, triaging lower acuity visits to avoid the need for in-person care, streamlining access to imaging and testing, and eliminating the need for unnecessary exposure to potential infection and COVID-19 transmission. In instances where patients were directed to come into the ED, physicians were able to provide a “warm handoff” to improve efficiency. This helped manage patient expectations, and the direct advice of the ED visit reassured them that the visit was warranted. Our findings suggest that the VED diverted low-acuity visits from the in-person ED waiting rooms and has the potential to lessen overcrowding.

Since the onset of the COVID-19 pandemic, numerous VEDs have been piloted and implemented out of necessity [13,15,26-29]. A systematic review of these programs found that virtual care during the COVID-19 pandemic was used as a means for pre-ED evaluation and screening, supplementing on-site care, postdischarge remote monitoring and treatment, care and resource coordination, and education. Therefore, most of these models were complementary workflows to supplement in-person ED care rather than a reimagination of the model itself [30]. In contrast to the SHSC VED, some VED used nurses or medical students for a pre-ED evaluation, where the ED nurse would perform the initial screening with advanced practice providers or physicians making final tele-triage decisions [31]. The SHSC VED differs in that it is staffed by an ED physician who may have more expertise and comfort to make decisions regarding the need for an in-person ED visit.

Our findings suggest that the VED is accepted by patients and physicians and is effective for certain patient presentations (such as orthopedic injuries, skin conditions, minor infections, and COVID-19 counseling). Similarly, a qualitative study of ED physicians found the VED can improve physician and patient experiences with emergency care [27]. Another systematic review pre-COVID-19 found many VED components were positive regarding technical quality and user satisfaction, but these studies were mainly regarding VEDs in small and rural hospitals to address infrequent but emergency situations requiring specialist care [32].

The SHSC model highlights the value of guiding patients regarding the decision for an in-person ED visit. Only one-quarter of survey respondents reported requiring an in-person assessment, with two-thirds reporting the VED eliminated the need for in-person care and added resource strain. This is comparable to the 17% of redirected patients in the pediatric VED model [16]. In another model with a phone emergency doctor service, nearly 3 of 4 (72.1%) patients were steered away from in-person emergency or clinic assessment [33]. Both of these VEDs were similar to the SHSC VED, where the patients self-triaged on the web and were then given virtual appointments with a family physician or an emergency physician.

This study provides initial insights into the implementation of a VED but has a few limitations to consider. We are only able to report on patient and physician experiences among those who agreed to be interviewed, and it is possible that those who did not complete a survey or participate in an interview had different experiences. Physician invitations came from JNH, the VED lead, which may have led to volunteer bias among these participants. Our data collection took place over a period when the VED was evolving to offer expanded capabilities, and assessing the impact of these changes was beyond the scope of this work. This study took place during the COVID-19 pandemic, and how these results translate as we transition to postpandemic times is unclear, as is the impact of the VED on clinical and economic outcomes. Finally, most patients who were engaged with the VED in this study were able to use technology with ease; however, our insights are limited as they relate to the impact of this model on digital equity. Further exploration is warranted (and ongoing with another research study locally) to ensure that the VED is not furthering the digital equity divide by only providing a service that a subset of patients can access. To make telehealth more accessible for older and more vulnerable patients, our findings indicate that having a simple interface and “live” administrative support was crucial in helping several patients access the VED. These essential design elements were highlighted in a review that indicated that to promote equity in virtual care and digital, it is essential to simplify interfaces and have supporting intermediaries to help patients engage with virtual care [34]. Future iterations must be carefully designed to reduce rather than enhance existing disparities in access to health care.

### Conclusions

As virtual care offerings become the standard of care, our results provide insights into why and how a VED provides immediate value to patients, is supported by ED physicians, and is an asset in facilitating care for lower acuity presentations that do not require an in-person visit. Future studies should focus on equity by understanding how VED access could be expanded to provide access for structurally marginalized groups. Research could also explore how a VED can create in-person capacity. From an implementation perspective, future work should explore the use of triage to guide patients to the most appropriate service location and modality close to home, thereby enhancing community access to rapid testing and investigations and integrating within the broader health care system. Permanent operational funding, enhanced and intuitive technology for seamless patient experiences, and ongoing provider and patient engagement will be critical to the long-term sustainability of virtual care in emergency medicine.

## Appendix

Multimedia Appendix 1 Patient survey for virtual emergency department evaluation.

Multimedia Appendix 2 Select survey results.

## Abbreviations

ED: emergency department
RE-AIM: Reach, Effectiveness, Adoption, Implementation, and Maintenance
REDCap: Research Electron Data Capture
SHSC: Sunnybrook Health Sciences Centre
VED: virtual emergency department

## Data Availability

This study generated both quantitative and qualitative data. The quantitative data set is not publicly available but is available from the corresponding author on reasonable request. The qualitative data are not available due to ethical constraints of the ethics board.
