# Supplementary material for: The Implementation of a Virtual Emergency Department: Multimethods Study Guided by the RE-AIM (Reach, Effectiveness, Adoption, Implementation, and Maintenance) Framework
Source: JMIR Form Res. 2023 Dec 5;7:e49786. doi: 10.2196/49786 (PMC10731546; doi:10.2196/49786)
Supplement: Multimedia Appendix 1 [file formative_v7i1e49786_app1.docx]

**Multimedia Appendix 1.** Patient survey for virtual emergency department evaluation.
