# Supplementary material for: The Implementation of a Virtual Emergency Department: Multimethods Study Guided by the RE-AIM (Reach, Effectiveness, Adoption, Implementation, and Maintenance) Framework
Source: JMIR Form Res. 2023 Dec 5;7:e49786. doi: 10.2196/49786 (PMC10731546; doi:10.2196/49786)
Supplement: Multimedia Appendix 2 [file formative_v7i1e49786_app2.docx]

**Multimedia Appendix 2.** Select survey results.

| **Question** | **Responses** | **%** |
| --- | --- | --- |
| Why did you choose to use the virtual urgent care service? | I felt like this was the most appropriate service to use  I tried to call my family doctor but could not make an appointment  I called my family doctor but it was too long until the next appointment  I called my family doctor and they directed me to call this service or sent me to the emergency department  I did not try to call my family doctor as this was more convenient  Other (please specify): | 35  14  14  9  8  19 |
| Would you have come in-person to the Emergency Department if virtual care was not available? | Definitely  Very likely  Unlikely  No | 39  31  17  13 |
| Thinking about this visit, what was the main reason why you accessed the virtual emergency department? | An accident or injury  A new health problem  An ongoing health condition or concern | 19  50  32 |
| Following my virtual urgent care visit | Did not need to access additional care  Was told to go to the emergency department right away  Was given an appointment at the emergency department  Was given an appointment with another health care provider  Was told to access care with my family physician | 32  20  7  25  17 |
| How would you rate your overall experience with virtual urgent care?, Mean (SD) | 1-10 | 9.1 (SD-1.5) |
| Overall, how satisfied were you with loading the software and logging into  the clinic? Mean (SD) | 1-10 | 9.2 (SD 1.5) |
| How would you rate the intensity or severity of your discomfort or symptom when you contacted the  virtual urgent care service? Mean (SD) | 1-10 | 6.6 (SD 2.1) |
| How would you rate the  intensity or severity of your  discomfort or symptom  between your virtual care  visit and now? Mean (SD) | 1-10 | 4.4 (SD 2.5) |
| The virtual urgent care service provided information that was useful for managing my care and treatment | Strongly Disagree  Disagree  Neither Agree nor Disagree  Agree  Strongly Agree | 5  3  10  26  57 |
| If the virtual urgent care service was not available, I would have gone to the  Emergency Department in person | Strongly Disagree  Disagree  Neither Agree nor Disagree  Agree  Strongly Agree | 8  10  21  32  30 |
| Receiving care through virtual visits has been better than the care that I have received in person | Strongly Disagree  Disagree  Neither Agree nor Disagree  Agree  Strongly Agree | 4  17  47  17  15 |
| I would use virtual urgent care again in  my healthcare | Strongly Disagree  Disagree  Neither Agree nor Disagree  Agree  Strongly Agree | 2  2  5  28  64 |
| I understand my health concern as  much as I can at this point in time | Strongly Disagree  Disagree  Neither Agree nor Disagree  Agree  Strongly Agree | 4  9  6  42  39 |
| Thinking about the health concern that brought you to the virtual urgent care service do you feel you have a plan you can follow? | Not at all  Somewhat  Moderately so  Very much so | 9  18  26  47 |
